# First Report on Neonatal Isolated Submandibular Suppurative Sialadenitis in Preterm Triplet Girls

**DOI:** 10.1155/crpe/4240104

**Published:** 2026-08-07

**Authors:** Patrick Morhart, Sergio Antonio Maravilla-Galdamez, Petros Strempas, Thomas Bollinger, Thomas Kienbaum, Heidi Weberruß

**Affiliations:** ^1^ Department of Neonatology, Childrens’ Hospital, Klinikum Bayreuth, Preuschwitzer Str. 101, 95445, Bayreuth, Germany; ^2^ Department of Microbiology, Klinikum Bayreuth, Preuschwitzer Str. 101, 95445, Bayreuth, Germany, klinikum-bayreuth.de

**Keywords:** neonatal infection, *S. aureus*, submandibular sialadenitis

## Abstract

Neonatal isolated submandibular suppurative sialadenitis (NISSS) is a rare condition. We present a case of NISSS caused by *Staphylococcus aureus* (*S. aureus*) in premature triplet girls born at 28 + 1 weeks of gestation. Mother’s milk culture tested positive for *S. aureus*, which is presumed to be the potential contributing factor of the infection. The first symptoms appeared in one of the girls at 31 days of age, followed by her sisters at 34 and 36 days of age, respectively. A search of the PubMed/Medline, ScienceDirect and Web of Science databases revealed that this is the first reported case of NISSS in triplets. We defined risk factors like an immature immune system, prolonged orogastric feeding and antidiuretic therapy for developing NISSS in neonates.

## 1. Introduction

Neonatal isolated submandibular suppurative sialadenitis (NISSS) is a rare distinct clinical entity [1–3]. Since Wells’ first report in 1975, a further 31 cases of NISSS have been reported in English literature, summarised in Table 1 [1, 2, 4–27]. According to Wells, NISSS was defined as swelling, erythema and induration over the submandibular gland with expression of purulent discharge [26]. In most patients, the infection was caused by *Staphylococcus aureus (S. aureus)*. Less frequent causes of infection were *Escherichia coli, Pseudomonas aeruginosa, Moraxella catarrhalis* and *Klebsiella pneumoniae* [1, 14]. Prematurity has been reported as a risk factor for developing NISSS in neonates. It is associated with an immature immune system, prolonged naso‐ or orogastric feeding, dehydration, supported ventilation and maternal mastitis [2, 3, 14, 25, 28]. Prolonged gastric feeding and the resulting hyposalivation and reduced ductal clearance are hypothesised to cause functional ductal obstruction and local inflammation [2, 29]. The typical manifestation of NISSS occurs within the first 2 weeks of life [11, 25].

**TABLE 1:** Course of NISSS in premature triplet girls.

	2nd triplet	1st triplet	3rd triplet
Onset of symptoms	31 days	34 days	36 days

Ultrasound examination	isolate submandibular swelling, increasing in size over 3 days, nascent abscess	isolated submandibular swelling, no abscess formation	isolated submandibular swelling, no abscess formation

Blood results	CRP 6.5 mg/L IL6 13.2 pg/mL	CRP 42.8 mg/L IL6 279 pg/mL	CRP 10.9 mg/L IL6 569 pg/mL

Antibiotic treatment	ampicillin/sulbactam (100/50 mg/kg) 31–40 days	ampicillin/sulbactam (100/50 mg/kg) 34–41 days	ampicillin/sulbactam (100/50 mg/kg) 36–43 days

Surgical intervention	35 days	—	—

## 2. Ethics Statement

Written informed consent for publication of this case report and any accompanying images was obtained from the patients’ parents. For this retrospective report, no ethical approval was asked for by the local ethics committee.

## 3. De‐Identification Statement

This case report presents de‐identified data of three patients. Identity of our patients cannot be traced back with this publication.

## 4. Case Presentation

We present data of triplet girls, dichorial triamniot, born at 28 + 1 weeks to a 29‐year‐old mother with good antenatal care. A caesarean section was performed due to premature rupture of the membranes and premature labour that did not respond to tocolysis. The mother received a single intramuscular administration of betamethasone 4 h prior to the caesarean section to prevent respiratory distress syndrome (RDS). The Apgar scores were 9/10/10 for the first child, 4/8/9 for the second child and 9/10/10 for the third child. The respective birth weights were 940, 890 and 840 g.

The first triplet received surfactant via the Less Invasive Surfactant Application (LISA) technique due to RDS on the second day of life. Furthermore, she was treated with spironolactone, hydrochlorothiazide, salbutamol and budesonide for symptoms of bronchopulmonary dysplasia from Day 16 to Day 53. The second triplet received LISA twice on the first day of life due to RDS. The third triplet underwent intubation due to hypercapnia on Day 2 and was given surfactant via an endotracheal tube after two LISA manoeuvres. Furthermore, the patient received intravenous paracetamol for a persistent ductus arteriosus (PDA) on Day 2, and ampicillin, gentamicin and vancomycin for suspected catheter sepsis from Days 11–12. She was also treated with spironolactone, hydrochlorothiazide, salbutamol and budesonide for symptoms of bronchopulmonary dysplasia from Days 16–53.

**FIGURE 1:**
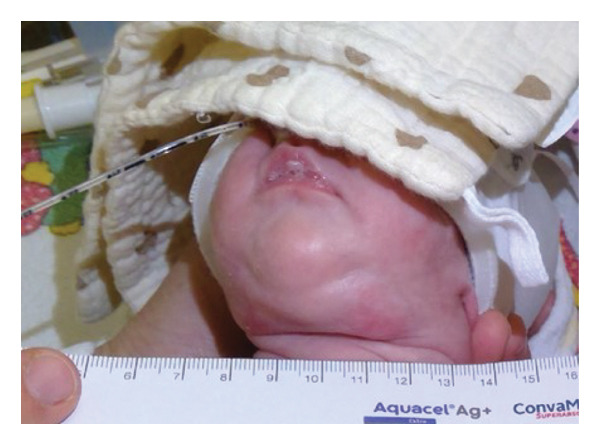
Neonatal patient with a bilateral submandibular swelling.

**FIGURE 2:**
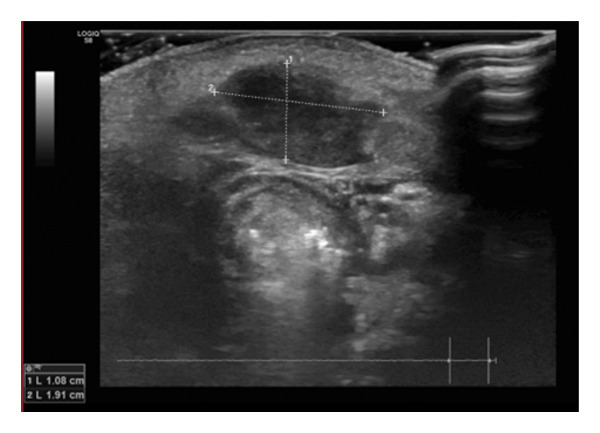
Ultrasound examination of the submandibular gland showing a longitudinally oval mass at the site of the submandibular gland, size 1.1–1.9 cm, anechoic in the center, with peripheral vascularity and an echogenic surrounding reaction.

On Day 31, the second triplet showed a tender submandibular swelling with local signs of inflammation (Figure 1). The patient was clinically in good condition, had no fever and was feeding well. Her white blood cell count (WBC) was 28.3/nL; she presented with lymphocytosis and 50% banded neutrophils. CRP and IL‐6 levels were 8.6 mg/L and 16.4 pg/mL, respectively. An ultrasound examination revealed a 1.1 × 1.9 cm submandibular mass, with no involvement of the parotid gland (Figure 2). We initiated antibiotic treatment with ampicillin/sulbactam (100/50 mg/kg) according to in‐house antibiotic stewardship. The antibiotic susceptibility test showed sensitivity for ampicillin/sulbactam. Surgical drainage was indicated after there was no reduction in the submandibular swelling after 72 h of antibiotic treatment and a nascent abscess diagnosed by ultrasound. One milliliter of purulent discharge was extracted. Microbiological cultures revealed *S. aureus* (methicillin‐susceptible, Panton‐Valentine leukocidin‐, toxic shock syndrome toxin‐1‐ and enterotoxin A‐D‐negative) to be the bacterial agent causing NISSS. A pharyngeal swab tested positive for *S. aureus*. PCR analysis for other potential viral or bacterial agents causing the infection tested negative. Ultrasound examination and laboratory results did not indicate a malignant lesion or further involvement of the sublingual or parotid gland or ductal system.

On Days 34 and 36 of life, the first and third triplet, respectively, presented with the same submandibular swelling as their sister. Ultrasound examinations of the swelling were consistent with NISSS. The first triplet presented with leucocytosis and elevated inflammatory markers (WBC 24.7/nL, CRP 42.8 mg/L and IL‐6270 pg/mL). The third triplet showed elevated CRP and IL‐6 levels (10.9 mg/L and 569 pg/mL, respectively). Both triplets tested positive for *S. aureus* in pharyngeal swabs; rectal temperatures were elevated (37.7°C and 38.1°C). Like their sister, both girls were in good clinical condition and feeding well. After 48 h of antibiotic treatment with ampicillin/sulbactam (100/50 mg/kg), a rapid reduction of the submandibular mass was observed in both girls, and no surgical intervention was indicated. The course of NISSS is displayed in Table 1.

In search of the potential source of the infection, mother’s milk tested positive for *S. aureus*, displaying the same antimicrobial susceptibility pattern as the pharyngeal swabs and purulent discharge from the second triplet. *S. aureus* was also found in swabs taken from the skin (axilla and groin) and nasopharynx of both parents.

To exclude other potential pathologies, immunoglobulin M (IgM) for the Epstein–Barr virus, hepatitis C and mumps was tested in the third triplet and found to be negative. CMV was tested for in the first and second triplets and was negative, too.

After 7–10 days of antibiotic treatment, the swelling had disappeared in all three patients. No re‐infection occurred until discharge. The parents of the triplets were treated for MSSA colonisation with the in‐house topical MRSA decolonisation regimen; the mother’s milk was pasteurised.

## 5. Discussion

To our knowledge, this is the first reported case of NISSS in triplets. A search of the PubMed/Medline, ScienceDirect and Web of Science databases (search term [neonatal isolated suppurative suppurative sialadenitis] AND [neonates], 01/11/2025) revealed that 31 cases have been published since Wells first described the condition in 1975 [26] (Table 2).

**TABLE 2:** Overview of studies reporting neonatal isolated submandibular sialadenitis (NISSS).

Author	Year	Patient’s age (days)	Gestational age (weeks)	Sex	Pathogen	Course
Said et al.	2008	10		no information given	n.n.	antibiotic treatment, full recovery

Bafaqeeh	1998	5	full term	no information given	Staph. aureus (MSSA)	antibiotic treatment, surgical drainage, full recovery

Banks et al.	1980	7	34	female	Staph. aureus (MSSA)	antibiotic treatment, full recovery

Bova and Walker	2000	8	full term	female	Staph. aureus (MSSA)	antibiotic treatment, full recovery

Cizmeci et al.	2014	6	36	male	n.n.	antibiotic treatment, full recovery

de Haan et al.	2003	8	27	male	Staph. aureus (MSSA)	antibiotic treatment, full recovery

Garavallo et al.	2001	14	30	female	Staph. aureus (MSSA)	antibiotic treatment, full recovery

Glibbery et al.	2021	7	37	male	Staph. aureus (MRSA)	antibiotic treatment, full recovery

Lindgren et al.	1998	25 43 37 13 20	32 25 23 31 29	Male female male male male	aseptic sialadenitis	antibiotic treatment, full recovery

Montero‐Lopez and Wintergerst	2021	8	full term	no information given	Staph. aureus (MSSA)	antibiotic treatment, full recovery

Mukhopadhyay et al.	2012	12	full term	male	Staph. aureus (MRSA)	antibiotic treatment, full recovery

Pereira et al.	2014	11	29	male	Staph. aureus (MSSA) *Klebsiella pneumoniae*	antibiotic treatment, full recovery

Ryan et al.	2014	20 16	full term full term	male	Staph. aureus (MSSA)	antibiotic treatment, full recovery

Salaria	2001		n.n.	n.n.	n.n.	n.n.

Shaughnessy et al.	2019	21	n.n.	n.n.	Staph. aureus (MSSA)	antibiotic treatment, surgical drainage, full recovery

Singh and Singhal	2004	40	25	male	Staph. aureus (MSSA)	antibiotic treatment, surgical drainage, full recovery

Takahashi et al.	2000	7 12	32 32	Male male	Staph. aureus (MRSA)	antibiotic treatment, full recovery

Kitapci and Calikoglu	2010	14	full term	male	n.n.	antibiotic treatment, full recovery

Ungkanont et al.	1998	8	34	male	Pseudomonas aeruginosa	antibiotic treatment, full recovery

Walsted	2001	14	n.n.	male	Staph. aureus (MSSA)	antibiotic treatment, full recovery

Weibel et al.	2005	10 13	full term full term	Male male	Staph. aureus (MSSA)	antibiotic treatment, full recovery antibiotic treatment, surgical drainage, full recovery

Wells	1975	14 6 6	32 35 31	Male n.n. female	Staph. aureus (MSSA)	antibiotic treatment, surgical drainage, full recovery antibiotic treatment, full recovery antibiotic treatment, full recovery

Almost all of the cases were caused by *S*. *aureus*. However, only one study found *S. aureus* as a suspected pathogen in mother’s milk [25]. In the present study, mother’s milk culture was tested positive for *S. aureus* and identified as a potential source of infection. As no molecular typing was performed to confirm strain identity between *S. aureus* found in purulent discharge, mother’s milk culture and pharyngeal swabs, we cannot clearly define mother’s milk as having caused NISSS in our patients. As alternative routes, horizontal transmissions in a setting where MSSA colonisation is common, like in neonatal intensive care units, could also be possible as well as transmission via parental colonization or endogenous colonization with ascending infection. However, contrary to most case reports, the patients in this study developed NISSS after the first 14 days of life. Due to the positive CMV status of the triplets’ mother, mother’s milk had to be pasteurised until 32 weeks of gestational age. The first affected second triplet exhibited NISSS symptoms 4 days after pasteurisation ceased. Contrary to most case reports, the patients in this study developed NISSS after the first 14 days of life. Due to the positive CMV status of the triplets’ mother, mother’s milk had to be pasteurised until 32 + 0 weeks of gestational age. The first affected second triplet exhibited NISSS symptoms 4 days after pasteurisation ceased. We therefore regard mother’s milk as a possible source of infection among other pathways stated.

Prematurity, immaturity of the immune system and prolonged naso‐ or orogastric feeding has been reported as potential risk factors for developing NISSS. These factors can also be applied to this case. Prolonged gastric feeding results in hyposalivation and reduced ductal clearance, causing functional ductal obstruction and local inflammation [2, 29]. The patients in this study received proper weight‐adapted enteral nutrition (mother’s milk and preterm formula). Their weight and diuresis were monitored daily with no indication of dehydration; thus, no weight loss was observed and diuresis of > 2–4 mL/kg/h.

Two of the triplets were treated with antidiuretic therapy with hydrochlorothiazide and spironolactone. Hydrochlorothiazide has been linked to xerostomia through a reduction in intracellular fluid and compensatory sympathetic activation, which results in hyposalivation in adults [30]. For spironolactone, a direct association to xerostomia could not be found in the literature. However, its diuretic effect is likely to increase dehydration. Furthermore, Leake and Leake [28] state that a moderate level of dehydration is sufficient to cause stasis and infection in the parotid gland. The same may hold true for the submandibular gland as well.

As in other studies, we did not observe sialoliths or anatomic malformations predisposing to NISSS. In addition, viral agents (CMV, EBV, hepatitis C and mumps) were ruled out as differential diagnoses [7, 14].

Ryan et al. and Wells et al. [15, 26] reported a case of NISSS in a pair of premature twins, but only one twin was diagnosed with NISSS caused by *S. aureus.* In the case report by Ryan et al. [15], one twin presented with *S. aureus* sepsis but not NISSS, while the other twin presented with NISSS. In our study, all three triplets presented with the same symptoms on subsequent days. All triplets had a positive pharyngeal swab for *S. aureus* and displayed the same antimicrobial susceptibility pattern.

No publication was found reporting a specific tropism for submandibular cells. Conversely, submandibular sialadenitis is less common than neonatal parotitis due to the structure of the submandibular gland, which consists of mucous and serous cells, with the components of the mucous cells providing bacteriostatic effects [26].

The course of our cases is comparable with NISSS cases reported by other authors. Hence, our patients were in good clinical condition, presenting no signs of sepsis. As in other studies, patients recovered shortly after the initiation of antibiotic treatment, with no recurrence observed [2, 8–11, 14, 15, 27].

### 5.1. Limitations

No blood cultures could be obtained in this study due to poor venous circulation in all three triplets. Since this report is a case study other than a controlled trial, diagnostic regimens were adapted to our patient’s condition and needs, not following a strict standardised protocol.

### 5.2. Patients’ Perspective

The patients’ clinical course has not been impaired by NISSS. They were treated with antibiotics without experiencing side effects. After cessation of the antibiotic therapy, no re‐infection occurred. In close communication with the triplets’ parents, ultrasound and laboratory findings were explained to create a transparent and trustworthy relationship.

## 6. Conclusion

In conclusion, this is the first case of NISSS reported in triplets. In accordance to other cases, *S. aureus* was identified as a pathogenic agent. We hypothesise an immature immune system in premature neonates, prolonged orogastric feeding and antidiuretic therapy as potential contributing factors that increase the risk for developing NISSS.

## Funding

The authors declare that no funding was received for the conduct of this study.

## Conflicts of Interest

The authors declare no conflicts of interest.

## Data Availability

The data supporting the findings of this study are available from the corresponding author upon reasonable request.
